# Reframing paucigranulocytic asthma through genetic endotyping: a hypothesis-generating focus on the 17q21 rs7216389 locus

**DOI:** 10.1097/ACI.0000000000001145

**Published:** 2026-03-27

**Authors:** Remo Poto, Rory Chan, Daniela Breda, Gilda Varricchi, Samuele E. Burastero

**Affiliations:** a Department of Translational Medical Sciences, University of Naples Federico II; b World Allergy Organization (WAO) Center of Excellence, Naples; c Istituti Clinici Scientifici Maugeri-IRCCS Scientific Institute of Telese Terme, Benevento, Italy; d University of Dundee, School of Medicine, Dundee, Scotland, UK; e Division of Immunology, Transplantation and Infectious Diseases, IRCCS San Raffaele Scientific Institute, Milan; f Center for Basic and Clinical Immunology Research (CISI), University of Naples; Federico II, Naples, Italy

**Keywords:** alarmins, rs7216389, severe asthma, thymic stromal lymphopoietin, type 2 inflammation

## Abstract

**Purpose of review:**

Non-T2 asthma is currently defined by missing parameters, such as low blood eosinophils and FeNO, rather than positively identifiable mechanistic features. This definition overlaps with paucigranulocytic asthma (PGA). However, T2-biomarkers fluctuate over time, especially during glucocorticoid therapy, leading to potential over-diagnosis of T2-low asthma. Advancing beyond traditional endotyping is required for precision medicine.

**Recent findings:**

Alarmin-driven asthma [interleukin (IL)-33, thymic stromal lymphopoietin (TSLP), IL-25] can drive inflammation even without high T2-markers, but these are difficult to measure clinically. Genetic testing, such as the rs7216389 SNP (17q21 locus, GSDMB/ORMDL3), offers a stable alternative. The T allele is linked to childhood-onset asthma, viral-induced alarmin release, and epithelial dysfunction. Notably, carriers of the T allele are more likely to respond to allergen immunotherapy (AIT). This genetic marker is not subjected to treatment-dependent modification and segregates with both T2-driven and alarmin-driven asthma.

**Summary:**

We propose that rs7216389 genotyping could be explored, within a treatable trait framework, to improve the mechanistic characterization of paucigranulocytic or low-biomarker asthma. While current data are associative, this one-time genetic assessment might contribute to research-driven stratification of “hidden” T2- or alarmin-leaning endotypes, potentially guiding the use of AIT and upstream biologics like anti-TSLP.

## INTRODUCTION

The advent of biologic therapies has transformed the clinical management of severe asthma by enabling targeted inhibition of Type 2 (T2) inflammation. Biologic agents directed against immunoglobulin E (IgE), interleukin (IL)-5/5Ra, IL-4Rα [13,14^▪^] and, more recently, upstream mediators like thymic stromal lymphopoietin (TSLP) have demonstrated robust efficacy across different phenotypes of the disease [15]. However, the traditional framework for identifying T2 inflammation, based on peripheral blood eosinophils, fractional exhaled nitric oxide (FeNO), and total serum IgE, is increasingly challenged by biological variability and limited sensitivity to airway-specific immune processes [16^▪^]. Factors such as smoking, viral infections, drugs, age, and time of sampling can significantly affect biomarker levels, leading to potential under-recognition of active T2 pathways [17]. Emerging data suggest that a substantial proportion of patients with so-called “low-T2 biomarker” asthma and airway hyperresponsiveness may nonetheless respond to antialarmin therapies [18], hinting at underlying pathogenic mechanisms that escape conventional detection. This recognition prompts a reconceptualization of asthma endotypes, shifting from a biomarker-centric to a mechanism-based approach, where intrinsic features such as epithelial dysfunction and innate immune activation become key components of disease stratification. Within this evolving paradigm, there is a pressing need for stable, noninvasive, and biologically informative markers that reflect airway pathobiology rather than fluctuating systemic readouts [2–9,12]. 

**Box 1:**
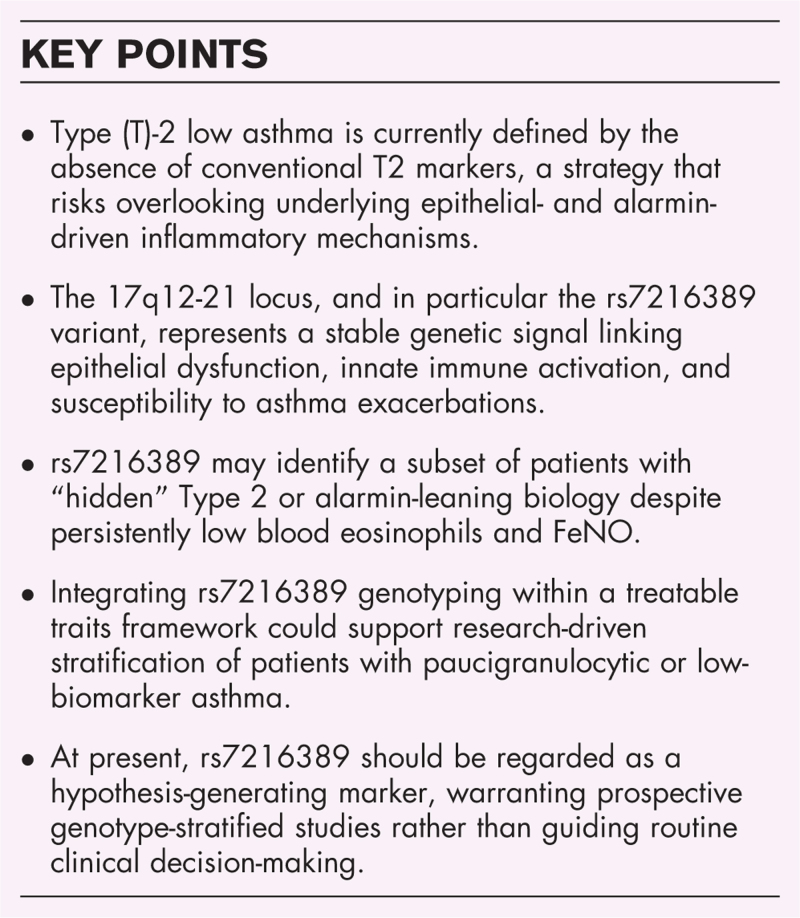
no caption available

### The 17q12-21 locus: a convergence point for genetic risk and epithelial dysfunction

Among the most consistently replicated genomic loci associated with asthma, the 17q12-21 region has been linked to early-onset wheeze, increased disease susceptibility, and a heightened risk of exacerbations [10^▪^,^19^^▪▪^,^20^]. This locus spans a cluster of genes involved in airway epithelial biology and immune regulation, including *ORMDL3* and *GSDMB*, which have been implicated in epithelial inflammation, barrier dysfunction, and airway remodeling [10^▪^]. The region is characterized by extensive linkage disequilibrium (LD), particularly in populations of European ancestry, suggesting tightly linked variants that modulate a shared pathogenic pathway [21^▪^].

Beyond 17q12-21, several other loci contribute to asthma susceptibility and to the spectrum of T2 and alarmin-related inflammation, including variants in *IL33*, *IL1RL1*, *TSLP*, *HLA-DQ*, *CDHR3*, and epithelial barrier genes such as filaggrin (FLG) [1]. These loci collectively support a model in which epithelial perturbation, innate immune activation, and allergen or viral exposures converge to shape distinct but overlapping endotypes. In this context, we focus on rs7216389 at 17q12-21 not as an isolated determinant of disease, but as an illustrative example of how a single, well characterized variant might be integrated into broader genetic endotyping strategies.

Genetic variation within 17q12-21 has also been associated with impaired antiviral responses, especially to rhinovirus, a major driver of asthma exacerbations [22^▪^]. These findings support the hypothesis that this locus contributes to a disease phenotype defined by epithelial dysfunction and dysregulated innate immunity. Notably, rs7216389, a SNP located at the distal boundary of the LD block [10^▪^] has emerged as a key variant of interest. In a *post hoc* analysis of a randomized controlled trial investigating house dust mite sublingual immunotherapy (SLIT), rs7216389 showed a significant gene-by-treatment interaction: patients homozygous for the T risk allele had a higher exacerbation rate under placebo and experienced a more pronounced clinical benefit from SLIT [11^▪▪^].

These results suggest that rs7216389 may serve not only as a marker of disease susceptibility but also as a predictor of therapeutic response, reinforcing its role in defining a genetically treatable Type-2 asthma endotype. In particular, given that house dust mite sensitization also impairs antiviral immunity, it can be speculated that house dust mite immunotherapy treatment interacts with 17q12-21 genotype through mechanisms related to antiviral immunity, including reduction of IL-33 production and restoring of the epithelial barrier.

### rs7216389: a boundary marker of genetic risk

The strategic position of rs7216389 within the 17q12-21 locus adds to its clinical and biological relevance. Linkage disequilibrium analysis of nine of the SNPs located in the 17q12-21 locus resulted in the pattern expected for a population of largely European descent: the SNPs upstream of and including rs7216389 showed a high pairwise linkage disequilibrium with a break of linkage downstream of rs7216389 [23]. Since rs7216389 is at the distal edge of the considered linkage disequilibrium block, the T allele's presence suggests high probability that upstream SNPs also carry the risk-associated alleles. That makes it informative and representative of the entire block in terms of both LD structure and disease association. The break of linkage downstream of rs7216389 which was observed, implies that SNPs beyond this point are less correlated, and the T allele at rs7216389 does not inform as well about alleles downstream. This structural characteristic makes this marker an informative and efficient proxy for genotyping the broader risk-associated architecture of the locus. Unlike complex cytokine assays or tissue-based biomarkers, genotyping this SNP is rapid, relatively inexpensive, and stable over time, making it an attractive candidate marker for research-oriented stratification in severe asthma [24]. Determination of the rs7216389 allele by standard PCR-based genotyping (for example, TaqMan allelic discrimination assays) is straightforward, using a noninvasive salivary swab for DNA sampling followed by DNA extraction and amplification with allele-specific primers, with allelic discrimination plots providing unambiguous genotype assignment, high reliability and ease of interpretation. Overall, the ability of rs7216389 to capture a shared pathogenic signature, spanning epithelial barrier dysfunction, innate immune dysregulation, and increased susceptibility to viral exacerbations, positions it as a promising candidate for integration into precision endotyping frameworks in asthma.

### Can rs7216389 help to identify noneosinophilic type 2 inflammation?

The genotyping of rs7216389 could, in principle, contribute to addressing a persistent clinical challenge: the identification of patients with T2 inflammation who lack traditional biomarkers such as blood eosinophils, elevated FeNO, or total/specific IgE; however, this remains an unproven and exploratory concept. These “low-T2 biomarker” phenotypes are increasingly recognized as potential responders to upstream biologic therapies, particularly those targeting alarmins like TSLP. However, the lack of accessible, validated markers for these endotypes hinders their identification in routine care.

We propose that rs7216389 may serve as a genetic surrogate for epithelial-driven T2 inflammation that is independent of eosinophilic activity. Association of the T risk allele with increased susceptibility to rhinovirus infections, overexpression of *ORMDL3* and *GSDMB*, and heightened risk of exacerbations is suggestive of a pathogenic pathway rooted in epithelial dysfunction and innate immune activation [25^▪^]. Unlike eosinophilic inflammation, which is readily measured in blood, alarmin-mediated responses remain elusive to standard clinical tests [26]. In this context, a stable, germline marker such as rs7216389 may provide a window into otherwise undetectable inflammatory activity.

This hypothesis reframes the role of rs7216389 from a static marker of disease risk to a dynamic tool for clinical stratification, particularly in those patients who fall outside conventional biomarker categories yet may still benefit from targeted intervention.

At present, no prospective study has validated rs7216389 as a standalone marker of noneosinophilic T2 inflammation, and any use of this genotype for patient stratification should be confined to research settings.

### Defining paucigranulocytic asthma in clinical practice

While paucigranulocytic asthma (PGA) is classically defined by sputum cytology showing low eosinophils and neutrophils, in real-world clinical settings, PGA is often operationally identified by persistently low FeNO (<25 ppb) and blood eosinophil counts (<150 cells/μl) when sputum induction is not available. To reduce the risk of misclassification due to transient biomarker suppression from glucocorticoids, infections, or other confounders, we recommend confirming low FeNO and blood eosinophil count values across multiple timepoints before considering a patient as PGA or T2-low. Importantly, these working definitions of PGA are pragmatic and partly extrapolated from clinical trial criteria rather than from PGA-specific validation studies. Likewise, the potential role of rs7216389 in this context is inferential, as no study has yet evaluated this genotype in rigorously defined PGA cohorts.

We propose that rs7216389 genotyping is best applied in patients who, despite serial biomarker assessments, persistently exhibit low T2 biomarkers. This approach enhances diagnostic confidence that the low T2 profile reflects a stable disease characteristic rather than a transient phenomenon.

### rs7216389 and paucigranulocytic asthma

Based on inflammatory cell characterization in induced sputum, asthma is categorized into eosinophilic, neutrophilic, mixed granulocytic, and PGA. The latter is characterized by the absence of elevated eosinophils or neutrophils in sputum and blood, is often unresponsive to conventional anti-inflammatory treatments and is frequently underrecognized [27]. PGA often presents later in life, with less atopy and lower IgE, contrasting with the early-onset, atopic, IgE-high asthma linked to rs7216389. Dissociation between the asthmatic phenotype and evidence of inflammation in the respiratory tract is the hallmark of this often “steroid insensitive” condition requiring alternative therapeutic strategies focused on structural airway alterations, neural dysregulation, smooth muscle hypertrophy, change of lifestyle factors, such as treatment of obesity, gastroesophageal reflux disease (GERD) or breathing patterns. Although PGA can transform into or develop from other phenotypes, 70% are stable over time [28].

Together, these observations support the hypothesis that PGA represents a distinct, inflammation-independent endotype of asthma, less influenced by genetic loci such as rs7216389 that predispose to immunologically mediated forms of the disease. Consequently, the presence or absence of this risk allele might, in future studies, help to explore differences between predominantly inflammatory and predominantly non-inflammatory asthma pathways. At this stage, such a role remains speculative and should be viewed as a hypothesis to be tested rather than as a tool for guiding personalized treatment strategies. While rs7216389 is unlikely to be a primary driver of PGA, it could play a role in cases where PGA transitions to an inflammatory phenotype (e.g., eosinophilic asthma) or *vice versa*, particularly in response to environmental factors like allergens or infections. For instance, if a patient with PGA develops an inflammatory trigger, rs7216389 could amplify the inflammatory response, potentially shifting the phenotype.

### Toward genotype-guided therapy in severe asthma

The rs7216389 asthma risk allele is strongly tied to inflammatory (particularly eosinophilic) asthma but is unlikely to play a major role in PGA, given PGA's non-inflammatory nature and distinct mechanistic drivers. In patients with ambiguous phenotypes or fluctuating biomarkers, genotyping might eventually be evaluated as one of several variables in exploratory models aiming to distinguish latent T2-driven disease from genuinely non-inflammatory pathways.

From a therapeutic standpoint, it is tempting to hypothesize that the rs7216389 T allele could modify responses to upstream biologics such as anti-TSLP or to allergen immunotherapy; however, no randomized trial has prospectively tested genotype-by-treatment interactions for this locus, and there is currently no evidence base to support rs7216389-guided therapy in clinical practice.

We propose a prospective evaluation of genotype-stratified therapeutic responses, particularly in PGA and low-biomarker asthma, to validate this approach.

**FIGURE 1:**
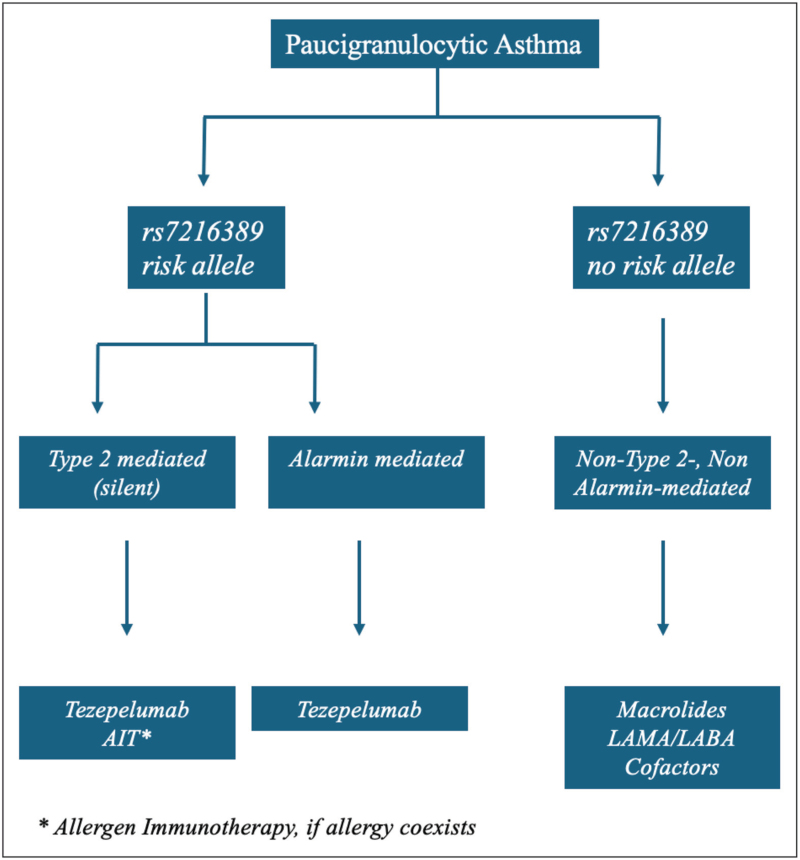
Conceptual model linking rs7216389 genotype to putative endotypic patterns and candidate therapeutic options in paucigranulocytic asthma (PGA). In this hypothesis-generating framework, the rs7216389T risk allele, which is associated with classical eosinophilic type 2 (T2) inflammatory profiles and elevated expression of ORMDL3/GSDMB, is used as a proxy for an underlying epithelial/alarmin axis that may be “silent” on routine biomarkers. This model illustrates how, in future research, genotype could be combined with biomarker-based T2 status to prioritize questions about eligibility and response to upstream biologics (such as tezepelumab) and, when sensitization co-exists, to disease-modifying treatments with allergen-specific immunotherapy (AIT). Absence of the risk allele is depicted as supporting a non-T2, nonalarmin-leaning phenotype, in which immunomodulatory and antiinfective interventions (macrolides), neural pathway modulation (long-acting muscarinic antagonists), bronchial thermoplasty, and interventions on lifestyle cofactors (obesity, GERD, breathing pattern) remain central. The figure is not intended to guide current clinical decision-making but to stimulate prospective, genotype-stratified studies.

Figure 1 illustrates the potential clinical utility of rs7216389 genotyping in PGA:

(1) T2-high (regardless of genotype): classical T2-high asthma, in which existing biomarker-driven algorithms for biologic eligibility remain the mainstay.
(2) T2-low, T allele present: a putative “hidden” T2- or alarmin-leaning phenotype in which upstream biologics (e.g. anti-TSLP) or AIT might, in future studies, be evaluated for differential efficacy according to genotype.
(3) T2-low, T allele absent: working model of non-T2, nonalarmin disease in which nonimmunologic strategies (macrolides, long-acting muscarinic antagonists, bronchial thermoplasty, and structured management of comorbidities such as obesity, GERD, or dysfunctional breathing) remain central. These groupings are not intended as treatment recommendations but as a research-oriented schema to prioritize questions for prospective, genotype-stratified studies.

This dissociation underscores the heterogeneity of asthma and highlights the need for precision medicine approaches tailored to specific endotypes. Future genetic studies should focus on PGA-specific loci, possibly involving airway smooth muscle, neuronal, or remodeling-related genes. To test our hypothesis, we look forward to seeing randomized controlled trials comparing rs7216389 genotype-driven treatment strategies to current standard of care.

In summary, rs7216389 at 17q12-21 exemplifies how a single, well characterized genetic variant might be integrated into modern treatable-trait thinking to move beyond fluctuating blood-based biomarkers, especially in patients with paucigranulocytic or low-biomarker asthma. At this stage, its role is purely investigational, but it provides a concrete starting point for designing genotype-stratified studies of upstream biologics and allergen immunotherapy, with the long-term goal of achieving more durable disease modification in appropriately selected patients.

## LIMITATIONS AND FUTURE DIRECTIONS

The conceptual framework proposed in this manuscript is subject to several important limitations. First, the effect sizes of asthma-associated variants at 17q12-21, including rs7216389, are modest at the individual-patient level and operate within a complex polygenic and environmental background. Second, most genetic data for this locus derive from children and from populations of European descent, limiting generalizability to adult-onset asthma and to diverse ancestries. Third, rs7216389 is in linkage disequilibrium with multiple functional candidates within the ORMDL3/GSDMB cluster, and association does not imply causation at the single-gene level. Fourth, there are currently no validated genotype cut-offs, clinical decision thresholds, or prospective trials testing rs7216389-by-treatment interactions for either biologics or allergen immunotherapy.

Taken together, these limitations reinforce that rs7216389 genotyping should not be used to guide individual treatment choices at present. Instead, it should be regarded as a tool for hypothesis generation that may, within carefully designed prospective and mechanistic studies, help to refine our understanding of epithelial- and alarmin-driven endotypes in severe asthma.

## Acknowledgements

*The authors thank Dr Gjada Criscuolo for her excellent managerial assistance.*

### Financial support and sponsorship

*The authors declare that no funding was received for this research.*

*Author contributions: R.P., R.C., D.B., G.V., and S.E.B. wrote the first draft of the manuscript. R.P., R.C., D.B., G.V., and S.E.B. reviewed and modified the manuscript. All authors contributed to the refinement of the final version and agreed to the decision to submit for publication.*

*Data availability statement: Data sharing is not applicable to this article as no datasets were generated or analyzed during the current study.*

### Conflicts of interest

*R.P. reports personal fees (talks) from AstraZeneca and GSK. R.C. reports institutional grants from Chiesi, AstraZeneca and GlaxoSmithKline for setting up and chairing the Scottish Airways Research Network; serving on an advisory board for AstraZeneca; personal fees (talks and drafting educational material) from AstraZeneca, personal fees (talks) from Chiesi, personal fees (talks) from Thorasys and personal fees (drafting educational material) from Vitalograph; support attending meetings from AstraZeneca, Chiesi, NIOX, Sanofi-Regeneron and Vitalograph. G.V. reports research support from AstraZeneca. SEB reports consulting for ALK Abellò. D.B. has no potential conflicts of interest to declare.*

## REFERENCES AND RECOMMENDED READING

Papers of particular interest, published within the annual period of review, have been highlighted as:

▪ of special interest
▪▪ of outstanding interest
